# Effects of divorce and widowhood on subsequent health behaviours and outcomes in a sample of middle-aged and older Australian adults

**DOI:** 10.1038/s41598-021-93210-y

**Published:** 2021-08-02

**Authors:** Ding Ding, Joanne Gale, Adrian Bauman, Philayrath Phongsavan, Binh Nguyen

**Affiliations:** 1 Prevention Research Collaboration, Sydney School of Public Health, Faculty of Medicine and Health, The University of Sydney, 6N69 Charles Perkins Centre (D17), Camperdown, NSW 2006 Australia; 2 Charles Perkins Centre, The University of Sydney, Camperdown, NSW Australia

**Keywords:** Epidemiology, Risk factors

## Abstract

Marital disruption is a common life event with potential health implications. We examined the prospective association of divorce/widowhood with subsequent lifestyles, psychological, and overall health outcomes within short and longer terms using three waves of data from the 45 and Up Study in Australia (T1, 2006–09; T2, 2010; T3, 2012–16). Marital status and health-related outcomes were self-reported using validated questionnaires. Nine outcomes were examined including lifestyles (smoking, drinking, diet and physical activity), psychological outcomes (distress, anxiety and depression) and overall health/quality of life. Logistic regression was adjusted for sociodemographic characteristics and baseline health outcomes. Of the 33,184 participants who were married at T1 (mean age 59.5 ± 9.3 years), after 3.4 years, 2.9% became divorced and 2.4% widowed at T2. Recent divorce was positively associated with smoking, poor quality of life, high psychological distress, anxiety and depression at T2. Similar but weaker associations were observed for widowhood. However, these associations were much attenuated at T3 (5 years from T2). Marital disruption in midlife or at an older age can be detrimental to health, particularly psychological health in the short term. Public awareness of the health consequences of spousal loss should be raised. Resources, including professional support, should be allocated to help individuals navigate these difficult life transitions.

## Introduction

Marital status and transitions may have important implications for health. It is generally well recognised that marriage can be protective for health and reduce morbidity and mortality^1^. Possible explanations for the beneficial effects of marriage may include a sense of greater social and financial support, overall healthier behavioural patterns, and self-selection where healthier individuals tend to marry^2^. In contrast, transitions out of marriage, such as becoming divorced or widowed, are stressful life events that have been associated with poor health and survival outcomes^1,3,4^. Marital disruption is a common life event: around 42% of marriages in England and Wales^5^ and about a third of marriages in Australia end in divorce ^6^. Between 1990 and 2010, the divorce rate in American adults aged 50 years and above doubled, implying a rising trend of “grey divorce”^7^. Even if a marriage survives without divorce, it will inevitably end with the death of a spouse, leaving the other one in widowhood, often for years. Several meta-analyses have shown that compared to married adults, divorced and widowed adults have a higher risk of mortality from all causes^1,8,9^ and specific causes including cardiovascular disease (CVD)^4^ and cancer^10^.

Contrary to the consistent observations about the disadvantage in health and survival following divorce or widowhood, the mechanisms underpinning these associations are less understood^11^. Amato’s Divorce-Stress-Adjustment Perspective postulates that the process of divorce leads to stressors, which in turn, increases emotional, behavioural and health risk. The risk, which could be either short- or long-term, may differ by individual characteristics and circumstances^12^. Within this model, psychological distress is a significant intermediate outcome of marital dissolution/bereavement, which may arise from financial and emotional challenges, and can lead to adverse health outcomes^11^. Another plausible intermediate outcome includes changes in lifestyle behaviours, which may be developed as a coping mechanism to deal with psychological distress, or a response to environmental, financial and other circumstantial changes. Such psychological and behavioural outcomes could in turn affect health, quality of life and wellbeing in the immediate-to-long term and longevity in the long term. To date, there has been limited longitudinal research on how divorce/widowhood affects both psychological wellbeing^13^ and lifestyle behaviours^14–16^. Furthermore, individuals respond and adjust to marital disruption differently^12^. Specifically men and women may have different coping strategies to psychological stressors^17^, and suffer from different consequences as a result of marital disruption^18^. For example, recent marital disruption has been associated with increased alcohol intake^19^ and decreased body mass index and vegetable intake in men^14^, and higher physical activity levels and a higher risk of smoking initiation/relapse in women^15^. Individuals with better socioeconomic status^20^ and social resources, such as supportive friends^21^, have also been reported to better cope with marital disruption.

With most marriages ending in divorce or widowhood, understanding the implications of marital disruption on health has important relevance to the life of many around the world. To date, most research has focused on the more “distal” outcomes, such as mortality. It is important to investigate modifiable and immediate outcomes on the pathways that lead to ill-health so that health deterioration may be prevented. It is also informative to examine whether such potential health effects persist over time. Such knowledge could improve the current understanding of the effects of major life events on health and inform interventions that aim to help individuals during marriage disruption. Moreover, previous research more commonly focused on divorce in younger populations, while the body of literature on divorce in older populations is much smaller despite the large proportion and the rising trend in “grey divorce”^7^. The objectives of this study were to examine the association of divorce and widowhood with subsequent changes in groups of selected outcomes: (1) health-related lifestyles, (2) psychological health, and (3) overall health and wellbeing, within both immediate and longer terms in middle-aged and older Australian adults.

## Methods

### Study population

Study participants were a subsample from the Sax Institute’s 45 and Up Study. Between February 2006 and December 2009, 267,153 adults aged 45 years and above from the state of New South Wales, Australia, submitted the baseline survey (T1, participation rate: 18%)^22^. Prospective participants were randomly sampled from the Services Australia (formerly the Australian Government Department of Human Services) Medicare enrolment database, which provides near complete coverage of the population. People aged 80 and over and residents of rural and remote areas were oversampled. In 2010, the first 100,000 respondents were invited to participate in a sub-sample follow-up study (T2): the Social, Economic, and Environmental Factor study (SEEF) (participation rate: 64.4%)^23^. Between 2012 and 2016, all living baseline participants were invited to participate in a full-sample follow-up, and 142,500 (53%) returned the survey (T3). Participants completed consent forms for all surveys. The baseline and full-sample follow-up data collection was approved by the University of New South Wales Human Research Ethics Committee (reference: HREC 05035) and the SEEF study by the University of Sydney Human Research Ethics Committee (reference: 10-2009/12187). The reporting of our analysis follows the Strengthening the Reporting of Observational Studies in Epidemiology (STROBE) guidelines (Supplementary file).

The study sample for the main analysis (Analysis 1) that focused on immediate outcomes included 33,184 men and women who reported to be in a married or cohabiting relationship at T1 and completed the marital status question at T2 (Supplementary Fig. 1). For those with additional follow-up data at T3, we conducted a subgroup analysis on the longer-term effects of marital disruption (Analysis 2) among those who reported to be married at T1, reported marital status at T2 and T3, and did not change marital status between T2 and T3 (Supplementary Fig. 2).

### Measures

Sex-specific baseline and full-sample follow-up questionnaires can be found at https://www.saxinstitute.org.au/our-work/45-up-study/questionnaires/. The SEEF questionnaire is included in Supplementary File.

#### Exposure variable

For the purpose of the study, both divorce/separation and widowhood were considered marital disruption^14,24^, but were considered as separate categories in the analysis because the two events usually happen at different stages in life within distinct circumstances and may have different implications on health^14^. For the purpose of the analysis, we combined those who were married and in a de facto relationship (living with a partner) together as “married”, because in Australia, those in a de facto relationship are considered legally similar to married couples^25^. In our sample, those in a de facto relationship are slightly younger than their legally married counterparts and account for 7% of the participants who were classified as “married” at T1. For Analysis 1, those who were married at both T1 and T2 were defined as “remained married”, those who were married at T1 but reported to be single, divorced or separated at T2 were defined as “recently divorced/seperated (‘divorced’ thereafter)” and those who were married at T1 but widowed at T2 were defined as “recently widowed”. For Analysis 2, those who reported to be in a married relationship at all three time points were defined as “continuously married”, and those who reported to be single, divorced or separated at T2 and T3 were defined as “remained divorced” and those who reported to be widowed at T2 and T3 were defined as “remained widowed”. Because the objective of Analysis 2 is to examine long-term implications of divorce and widowhood, we focused the analysis on those whose marital status remained the same between T2 and T3, and excluded those who became divorced or widowed between T2 and T3 due to the recency of events (n = 1768), those who remarried/re-partnered between T2 and T3 due to the lack of consistent exposure (n = 145), and those who changed between divorced and widowed because the events were difficult to interpret (n = 27).

#### Outcome measures

We examined nine self-reported outcome variables in three categories: (1) health-related lifestyles: smoking, alcohol consumption, diet and physical activity; (2) psychological outcomes: psychological distress, anxiety and depression; (3) overall health and wellbeing: self-rated health and quality of life. Responses were coded as 1 for being “at risk” and 0 for “not at risk”, as described in Table 1.

**Table 1:** Measures and scoring of health-related outcomes in the 45 and Up Study.

Health-related outcomes	Measures	Scoring methods (1 = at risk; 0 = not at risk)
**Lifestyle behaviours**		
Smoking	“Have you ever been a regular smoker?” and “Are you a regular smoker now?”	1 = “yes” to both questions
Alcohol consumption	“About how many alcoholic drinks do you have each week? (one drink = a glass of wine, middy of beer or nip of spirits)”	1 = more than 14 drinks per week (exceeding the current Australian Guidelines^a^ on alcohol consumption)
Fruit and vegetable intake	A validated questionnaire^b^ on total serves of fruit and vegetables (raw or cooked) usually consumed each day	1 = not consuming the recommended two serves of fruit and five serves of vegetables per day^c^
Physical activity	The validated Active Australia Questionnaire,^d^ which asked about the total time spent on walking, moderate-, and vigorous-intensity physical activity (in bouts of at least 10 min) in the last week. Total physical activity was calculated by summing the three types of activities with vigorous activity weighted by two	1 = not meeting the lower threshold of the recommended amount (150 min/week) based on Australia’s Physical Activity and Sedentary Behaviour Guidelines^e^
**Psychological outcomes**		
Psychological distress	Kessler 10 (K10),^f^ a validated and widely used 10-item questionnaire to measure general psychological distress experienced in the last 4 weeks. Score ranges from 10 to 50 with higher scores representing higher levels of distress	1 = high-to-very high psychological distress (K10 ≥ 22)
Anxiety	“Has a doctor ever told you that you have anxiety?”	1 = “yes”
Depression	“Has a doctor ever told you that you have depression?”	1 = “yes”
**Overall health and wellbeing**		
Self-rated health	“In general, how do you rate your overall health?” from the Medical Outcomes Study 12-Item Short-Form Health Survey (SF-12)	1 = “fair” or “poor”
Quality of life	“In general, how do you rate your quality of life?”	1 = “fair” or “poor”

^a^Australian Government National Health and Medical Research Council. Australian guidelines to reduce health risks from drinking alcohol, 2009.

^b^Evaluation of short dietary questions from the 1995 National Nutrition Survey; Australian Food and Nutrition Monitoring Unit. Canberra: Health and Aged Care; 2001.

^c^Australian Government Department of Health and Ageing. Eat for health: Australian Dietary Guidelines, 2013.

^d^Brown WJ, Burton NW, Marshall AL, Miller YD. Reliability and validity of a modified self-administered version of the Active Australia physical activity survey in a sample of mid-age women. Aust N Z J Public Health 2008; 32(6): 535–41.

^e^The Department of Health Australian Government. Australia's Physical Activity and Sedentary Behaviour Guidelines, 2014.

^f^Andrews G, Slade T. Interpreting scores on the Kessler Psychological Distress Scale (K10). Aust N Z J Public Health 2001; 25(6): 494–7.

#### Covariates and effect modifiers

The following variables were selected as covariates: age (continuous), sex, educational attainment (up to 10 years, high school/diploma/trade, university), residential location (major city vs regional/remote, based on the Accessibility Remoteness Index of Australia^26^), country of birth (Australia vs overseas) and follow-up time. Specifically, we selected education, rather than income, as a socioeconomic indicator, because previous research repeatedly concluded that education generally has the strongest effects on health behaviors^27^, and it has nearly complete data in the 45 and Up Study. Therefore, it has been consistently recommended as a stable and reliable socioeconomic indicator for the current cohort^28,29^.

In addition, several variables were selected as potential effect modifiers based on evidence from previous studies, including: age categories, sex, educational attainment and social support^14,17,21,30–32^. Based on previous evidence suggesting that friends’, rather than family’s support buffers health deterioration following marriage disruption^21^, we used one question from the Duke Social Support Index^33^ to measure social support outside of family. The question asks about the number of people outside of home within one hour of travel one can depend on or feel close to. Based on previous investigation in the SEEF study, this single question had the most consistent association with psychological distress across sex and age categories and was therefore chosen as an indicator for social support^34^. Responses were dichotomised at the median into low (0–4 people) and high (5 + people).

### Statistical analysis

Baseline sociodemographic characteristics and health-related outcomes of the three marital transition groups were compared using ANOVA and *χ*^2^ tests. For Analysis 1, those who remained married served as the reference category when comparing outcomes with those who became divorced and widowed. For Analysis 2, those who were “continuously married” between T1 and T3 served as the reference category when comparing outcomes with those who “remained divorced” or “remained widowed”. Separate binary logistic regression models were fitted for each dichotomous outcome, adjusted for all covariates and the value of each outcome at T1. Effect modification was tested by including a multiplicative interaction term in the adjusted model followed by a likelihood ratio test. Given the small amount of missing data (< 8%), we used missingness as a category for analysis. Considering that people who became divorced or widowed by T2 may be at a higher risk for death or loss to follow up by T3, posing threats to selection bias, we conducted additional analyses outlined in Supplementary file (page 5 “Methodological supplement”). All statistical analyses were conducted using SAS 9.4 and significance levels were set at *p* < 0.05.

### Ethical approval

Approved by the University of New South Wales Human Research Ethics Committee (reference: HREC 05035) and the SEEF study by the University of Sydney Human Research Ethics Committee (reference: 10-2009/12187).

## Results

### Baseline descriptive statistics

Of the 33,184 participants who were married at baseline (T1, 2006–2009), after a mean follow-up time of 3.35 (standard deviation [SD] = 0.95) years, 31,760 (95.7%) remained married at the first follow-up (T2, 2010), 616 (2.9%) became divorced and 808 (2.4%) became widowed. At T1, compared with those who remained married, those who recently divorced were younger and had slightly higher levels of education, were less likely to live in major cities and more likely to be born overseas. On the contrary, those who recently widowed were much older, predominantly females, had lower educational attainment, and were less likely to live in major cities (Table 2).

**Table 2:** Baseline (T1, 2006–2009) characteristics of participants by marital status at T2 (2010).

Characteristics	Overall (n = 33,184)	Remained married (n = 31,760)	Recently divorced (n = 616)	Recently widowed (n = 808)
	n (%)	n (%)	n (%)	n (%)
**Sociodemographic characteristics**^**a**^				
Age: Mean (SD)	59.5 (9.30)	59.3 (9.15)	55.9 (8.67)	68.8 (10.60)
Sex (female)	17,799 (53.6)	16,898 (53.2)	328 (53.3)	573 (70.9)
**Education**				
Low (up to 10 years)	9235 (28.2)	8732 (27.8)	145 (23.7)	358 (45.3)
Mid (high school/diploma/trade)	14,113 (43.0)	13,531 (43.1)	281 (46.0)	301 (38.1)
High (university degree)	9452 (28.8)	9136 (29.1)	185 (30.3)	131 (16.6)
Living in major cities	16,335 (50.1)	15,696 (50.3)	276 (45.8)	363 (45.5)
Born overseas	7667 (23.1)	7323 (23.1)	157 (25.5)	187 (23.1)
**Baseline health behaviours and outcomes**^**b**^				
Fair/poor self-rated health	1997 (6.2)	1874 (6.1)	63 (10.5)	60 (7.8)
Fair/poor quality of life	1364 (4.3)	1240 (4.1)	58 (9.8)	66 (8.7)
High Kessler 10 score (K10 ≥ 22)	1378 (4.5)	1291 (4.4)	54 (9.4)	33 (4.9)
Anxiety diagnosis	1745 (7.9)	1661 (7.8)	51 (13.3)	33 (7.6)
Depression diagnosis	2606 (11.8)	2487 (11.7)	82 (21.4)	37 (8.5)
Smoking	1673 (5.0)	1574 (5.0)	63 (10.2)	36 (4.5)
Alcohol ≥ 14 serves/week	6566 (20.0)	6290 (20.1)	137 (22.6)	139 (17.6)
Physical inactivity	5674 (17.5)	5377 (17.3)	129 (21.7)	168 (21.8)
Insufficient fruit and vegetable intake	24,840 (76.7)	23,771 (76.7)	492 (81.7)	577 (74.2)

T1: baseline data collection (2006–09), T2: first follow-up: 2010.

Remained married: married at T1 and T2; Recently divorced: married at T1 and divorced/separated at T2; Recently widowed: married at T1 and widowed at T2.

^a^ Sociodemographic characteristics differed significantly by marital status at T2 (*p* < 0.001) for all variables except for “born overseas” (*p* = 0.227).

^b^All baseline health behaviour and outcome variables differed significantly by marital status (*p* < 0.01).

At T1, compared with those who remained married, those who recently divorced had around twice the prevalence of fair/poor self-rated health and quality of life, high psychological distress, anxiety, depression, and reported smoking. They also had a slightly higher prevalence of high alcohol consumption, physical inactivity, and insufficient fruit and vegetable intake. Those who were recently widowed had a higher prevalence of fair/poor self-rated health and quality of life, high psychological distress, and physical inactivity, but lower prevalence of depression, smoking, at-risk alcohol consumption, and insufficient fruit and vegetable intake.

### Analysis 1: short-term health outcomes following marital disruption

After adjusting for sociodemographic characteristics, health-related outcomes at T1, and follow-up time, those who recently divorced had much higher odds of fair/poor quality of life (Odds Ratio [OR] = 2.98), high psychological distress (OR = 2.78), smoking (OR = 2.40), anxiety (OR = 2.23) and depression (OR = 2.92) at T2 (Table 3). The associations of divorce with fair/poor self-rated health (OR = 1.22), high alcohol consumption (OR = 1.12), physical inactivity (OR = 1.04) and insufficient fruit and vegetable consumption (OR = 1.25) were non-significant. For nearly all outcomes, adjusting for covariates attenuated the associations. When comparing those who were recently widowed with those who remained married, based on adjusted analysis, recent widows had higher odds of fair/poor quality of life (OR = 1.80), high psychological distress (OR = 1.92), anxiety (OR = 1.55), depression (OR = 2.11), smoking (OR = 2.51), and insufficient fruit and vegetable consumption (OR = 1.60). Recent widows also had a marginally lower prevalence of high alcohol consumption at T2 (OR = 0.75).

**Table 3:** Odds ratios for the associations of marital disruption which occurred between T1 (2006–2009) and T2 (2010) with health-related outcomes at T2 (n = 33,184).

Outcomes	Recently divorced (Reference: remained married)		Recently widowed (Reference: remained married)	
	Unadjusted	Adjusted^a^	Unadjusted	Adjusted^a^
Fair/poor self-rated health	**1.35 (1.06, 1.72)**	1.22 (0.92, 1.62)	**1.54 (1.25, 1.88)**	0.98 (0.77, 1.23)
Fair/poor quality of life	**3.05 (2.42, 3.85)**	**2.98 (2.28, 3.88)**	**2.75 (2.22, 3.42)**	**1.80 (1.40, 2.31)**
High Kessler 10 score (K10 ≥ 22)	**3.11 (2.43, 3.97)**	**2.78 (2.11, 3.67)**	**1.80 (1.38, 2.34)**	**1.92 (1.43, 2.57)**
Anxiety	**2.30 (1.61, 3.28)**	**2.23 (1.56, 3.19)**	1.45 (0.98, 2.14)	**1.55 (1.04, 2.31)**
Depression	**3.07 (2.17, 4.35)**	**2.92 (2.06, 4.14)**	**1.85 (1.28, 2.67)**	**2.11 (1.45, 3.08)**
Smoking	**2.83 (2.15, 3.71)**	**2.40 (1.51, 3.81)**	1.22 (0.87, 1.72)	**2.51 (1.48, 4.26)**
Alcohol ≥ 14 serves/week	**1.21 (1.00, 1.47)**	1.12 (0.85, 1.47)	**0.66 (0.54, 0.82)**	0.75 (0.57, 1.00)
Physical inactivity	1.05 (0.85, 1.30)	1.04 (0.84, 1.30)	**1.38 (1.16, 1.64)**	1.10 (0.92, 1.33)
Insufficient fruit and vegetable intake	**1.37 (1.12, 1.68)**	1.25 (1.00, 1.56)	1.13 (0.95, 1.33)	**1.60 (1.33, 1.92)**

T1: baseline data collection (2006–09), T2: first follow-up: 2010. Boldface indicates statistical significance at *p* < 0.05.

Remained married: married at T1 and T2; Recently divorced: married at T1 and divorced/separated at T2; Recently widowed: married at T1 and widowed at T2.

^a^Adjusted for the outcome value at T1, age, sex, educational attainment, residential location (major cities vs regional/remote), country of birth (Australia vs overseas), and follow-up time between T1 and T2.

Several sociodemographic characteristics seemed to have moderated the association between marital disruption and short-term health outcomes (Table 4). Specifically, the association between divorce and quality of life was the strongest (OR = 4.91) in the oldest group (75 + years), but the association between widowhood and quality of life was the strongest (OR = 3.35) in the youngest group (45–59 years). On the other hand, the associations of both divorce and widowhood with psychological distress were the strongest in the youngest group (OR = 2.98 and 3.53, respectively). The association between divorce and high psychological distress was stronger among those with lower education attainment (OR = 2.96, up to 10 years education; OR = 3.06, high school/diploma) but the association between widowhood and psychological distress was stronger among those with high educational attainment (OR = 4.20). The association of divorce with depression was much stronger in men (OR = 4.59) than women (OR = 1.60) but the association of widowhood was similar by sex. While there was no significant association between divorce and alcohol consumption, recent widowhood seemed to reduce the risk of high alcohol consumption among women (OR = 0.53). Finally, while there was no observed association between divorce and physical activity, widowhood was significantly associated with insufficient physical activity in those with a medium level of educational attainment only (OR = 1.46).

**Table 4:** Associations of marital disruption which occurred between T1 (2006–2009) and T2 (2010) with health-related outcomes at T2, stratified by statistically significant effect modifiers.

	Recently divorced (Reference: remained married)	Recently widowed (Reference: remained married)	*P for interaction*
**Outcome: Fair/poor quality of life**			
*Stratified by age categories (years)*			0.003
45–59	2.84 (2.08, 3.89)	3.35 (2.09, 5.38)	
60–74	1.74 (0.95, 3.20)	2.00 (1.37, 2.92)	
75+	4.91 (1.96, 12.31)	1.08 (0.71, 1.66)	
**Outcome: High Kessler 10 score (K10 ≥ 22)**			
*Stratified by age categories (years)*			0.003
45–59	2.98 (2.20, 4.04)	3.53 (2.25, 5.53)	
60–74	1.59 (0.73, 3.47)	1.41 (0.86, 2.33)	
75+	1.65 (0.33, 8.16)	0.90 (0.47, 1.73)	
*Stratified by educational attainment*			0.048
Low (up to 10 years)	2.96 (1.80, 4.87)	1.65 (1.09, 2.51)	
Mid (high school/diploma/trade)	3.06 (2.08, 4.49)	1.32 (0.77, 2.25)	
High (degree)	1.48 (0.73, 2.98)	4.20 (2.26. 7.81)	
**Outcome: Depression diagnosis**			
*Stratified by sex*			0.017
Male	4.59 (2.94, 7.17)	1.85 (0.89, 3.86)	
Female	1.60 (0.90, 2.86)	2.01 (1.30, 3.11)	
**Outcome: High alcohol consumption**			
*Stratified by sex*			0.033
Male	1.14 (0.79, 1.63)	1.14 (0.75, 1.74)	
Female	1.17 (0.76, 1.79)	0.53 (0.36, 0.79)	
**Outcome: Insufficient physical activity**			
*Stratified by educational attainment*			0.042
Low (up to 10 years)	0.72 (0.45, 1.14)	0.98 (0.75, 1.29)	
Mid (high school/diploma/trade)	1.05 (0.76, 1.46)	1.46 (1.09, 1.95)	
High (degree)	1.14 (0.76, 1.71)	0.68 (0.40, 1.16)	

T1: baseline data collection (2006–09), T2: first follow-up: 2010.

Remained married: married at T1 and T2; Recently divorced: married at T1 and divorced/separated at T2; Recently widowed: married at T1 and widowed at T2.

### Analysis 2: long-term health outcomes following marital disruption

After an additional five years (mean = 4.98, SD = 0.53) of follow-up, a total of 21,605 participants reported marital status at the second follow-up (T3, 2012–2016) and did not change relationship status between T2 and T3, so that consistent relationship patterns could be determined and long-term outcomes of marital disruption that occurred between T2 and T3 could be examined. Of this subgroup of participants, 20,900 were consistently married (96.7%), 270 (1.25%) remained divorced and 435 (2.01%) remained widowed. The comparison of baseline characteristics across the three groups remained similar to that from Analysis 1 (Supplementary Table 1). When adjusting for sociodemographic characteristics, health-related outcomes at T1, and follow-up time, those who remained divorced still had higher odds for most adverse health outcomes compared with those who were consistently married (Table 5), but the associations were much weaker compared with those observed in Analysis 1, and only reached statistical significance for insufficient fruit and vegetable consumption (OR = 1.55). Compared with those who were consistently married, those who remained widowed did not have consistently higher odds for adverse health outcomes and none of the associations was statistically significant. We did not find significant effect modification by age, sex, educational attainment, or social support.

**Table 5:** Odds ratios for the associations of marital disruption which occurred between T1 (2006–2009) and T2 (2010) with health-related outcomes at T3 (n = 21,605).

Outcomes	Remained divorced (Reference: continuously married)		Remained widowed (Reference: continuously married)	
	Unadjusted	Adjusted^a^	Unadjusted	Adjusted^a^
Fair/poor self-rated health	1.20 (0.79, 1.82)	1.01 (0.63, 1.62)	**1.70 (1.27, 2.27)**	1.20 (0.86, 1.66)
Fair/poor quality of life	1.54 (0.96, 2.47)	1.31 (0.79, 2.19)	**1.53 (1.05, 2.23)**	0.70 (0.46, 1.07)
High Kessler 10 score (K10 ≥ 22)	**1.71 (1.12, 2.61)**	1.41 (0.89, 2.24)	1.14 (0.75, 1.71)	0.79 (0.51, 1.21)
Anxiety^b^	1.66 (0.84, 3.27)	1.64 (0.83, 3.24)	1.06 (0.52, 2.16)	1.15 (0.56, 2.38)
Depression^b^	1.48 (0.58, 3.10)	1.48 (0.65, 3.39)	0.99 (0.39, 2.06)	1.07 (0.46, 2.46)
Smoking	**2.54 (1.50, 4.30)**	1.48 (0.72, 3.05)	1.24 (0.69, 2.21)	1.65 (0.74, 3.67)
Alcohol ≥ 14 serves/week	1.23 (0.92, 1.65)	1.03 (0.7, 1.52)	0.63 (0.47, 0.84)	0.78 (0.54, 1.13)
Physical inactivity	1.06 (0.85, 1.48)	1.06 (0.75, 1.50)	**1.42 (1.12, 1.81)**	0.95 (0.74, 1.24)
Insufficient fruit and vegetable intake	**1.52 (1.12, 2.07)**	**1.55 (1.12, 2.15)**	0.81 (0.66, 1.00)	1.09 (0.87, 1.37)

T1: baseline data collection (2006–09); T2: first follow-up: 2010; T3: second follow-up: 2012–16. Boldface indicates statistical significance at *p* < 0.05.

Continuously married: married at T1, T2 and T3; Remained divorced: married at T1 and divorced/separated at T2 and T3; Remained widowed: married at T1 and widowed at T2 and T3.

^a^Adjusted for the outcome value at T1, age, sex, educational attainment, residential location (major cities vs regional/remote), country of birth (Australia vs overseas), and follow-up time between T1 and T3.

## Discussion

This study examined the short- and long-term health outcomes following divorce and widowhood in a large population-based Australian sample of older men and women. The findings revealed strong and adverse short-term effects of marital disruption on health outcomes, particularly within the psychological health domain. These effects seemed to attenuate in the longer term.

A number of studies have examined the associations between marital status or marriage disruption and health, with relatively consistent findings suggesting a protective effect of marriage, and respectively detrimental effects of marital disruption. For example, systematic reviews and meta-analyses have consistently found an elevated risk of all-cause mortality in adults who are divorced^35^ or widowed^1,8,9^, and the effects seemed to be mostly consistent across countries and geographic areas^1,9^. Wong et al. extended the outcomes for CVD and found similar associations between marital status and CVD events and mortality^4^. Our current study has extended previous research by examining a broad range of relatively proximal outcomes, and in a population-based sample ranging from middle age to the “oldest old”. Examining proximal outcomes could help understand the potential mechanisms (e.g., psychological distress, unhealthy lifestyles) for the observed association between marital disruption and distal endpoints, such as mortality. Understanding the potential mechanisms has been considered an important research agenda for future studies^35^. Involving a large sample with a broad age range allows us to examine the effect of marital disruption at different life stages, including the less researched transitions, such as divorce at an old age (grey divorce)^7^ and widowhood at a younger age.

To date, several proposed mechanisms might explain the health disparities by marital status^4^. The predominant debate has centered around social selection versus causation^36^. While selection theory suggests that people with poorer health are less likely to enter or maintain long-term partnerships^4,36^, social causation theory postulates that marriage and partnership benefit individuals’ health through spousal support, companionship and financial stability^36–38^. Within the causation theory framework, it has been proposed that the stress related to spousal loss could affect physical, mental, emotional and behavioural health^4,36,38–40^. In the current study we tested various components of these theories by: (1) comparing baseline characteristics of participants with different marriage transitions, (2) adjusting for potential confounders that could have caused self-selection into maintained partnership, such as socioeconomic status, and (3) comparing between those who have divorced and widowed, which involve different levels of self-selection.

Based on the baseline comparison of participants with different marital transition categories, those who became divorced at T2 appeared to be distinctly different from the other categories at T1: they had around twice the prevalence of fair/poor self-rated health and quality of life, high psychological distress, anxiety, depression, and current smoking, compared with those who remained married between T1 and T2. In most cases, they had much worse health risk profiles than those who became widowed, despite the latter being significantly older. Such observations may provide supportive evidence for the social selection theory. However, given that the deterioration of marriage is a gradual process, which started from the time when couples still lived together^12^, a dysfunctional relationship could have adversely affected physical and mental health years before divorce or separation formally took place^12^. In both short- and longer-term analyses, adjusted associations were much attenuated from the unadjusted associations, suggesting that the potential characteristics underlying social selection to marriages, such as socioeconomic status, may have partially contributed to the observed “marital disruption effects”. However, the adjusted associations remained strong in most cases, implying the plausibility for a causal relationship. Finally, we found generally similar patterns of associations for divorce and widowhood; if social selection was the sole explanation for the detrimental health effects of marital disruption, then one should expect strong effects of divorce but much weaker-to-no effects of widowhood, because spousal death is usually beyond the control of the surviving spouse^24^.

As an attempt to explore different mechanistic pathways, assuming that marital disruption is causally linked to health deterioration, we tested several domains of health outcomes: health-related lifestyle behaviours, psychological outcomes, and overall health and wellbeing. Our findings suggest that most of the observed “marital disruption effects” occurred within the psychological domain, with divorce and widowhood triggering initial elevations in psychological distress, anxiety and depression. The much higher odds of smoking among those who recently divorced or widowed, similar to findings from a previous study^15^, could also be stress-related^41^. Contrary to previous studies^14,15,42^, we found no overall associations between marital disruption and physical activity or alcohol consumption. We did, however, find a positive association between divorce/widowhood and insufficient fruit and vegetable consumption. Based on a small number of studies, vegetable consumption seemed to decline in men^14^ and women^15^ following divorce and widowhood, and the literature has cited a lack of food preparation skills among men^14^ and meal skipping as a grief reaction among women^15^. Finally, within the overall health and wellbeing domain, recently divorced and widowed individuals suffered from worsening quality of life but not self-rated health. This could be because the self-rated health question focuses on the physical manifestation of health while the quality of life question holistically captures physical, mental, emotional and other aspects of health, which are more likely to be influenced by marital disruption.

An interesting finding is that although marital disruption seemed to have a detrimental effect on various health outcomes in the short-term, after a further five years of follow-up, the effects were attenuated, and in some cases, disappeared. These findings confirmed the “divorce-stress-adjustment perspective”^12^, which postulates that marital disruption led to multiple stressors (e.g., loss of custody of children, economic decline), which, in turn, lead to negative emotional, behavioural and health outcomes. The process of “adjustment” takes time, and its severity and duration differ by individual characteristics^12^. Previous research found a similar “time effect” (where the negative consequences of marital disruption was attenuated over time) with depression^43^, first-time myocardial infarction^44^ but mixed results with mortality^40,45^. However, it is important to distinguish our study from those with morbidity or mortality endpoints, which take longer to manifest. Given that outcomes in our study are conceptually proximal, and that most people have the psychological resilience to eventually recover from marriage disruption^46^, we could expect *on average* a stronger effect in the short-term than the long-term.

However, it is important to acknowledge individual differences in resilience to stressful transitions like divorce and widowhood^46^. We have tested for several potential effect modifiers and found several outcome-specific interactions. For example, overall, younger participants (aged 45–59 years at T1) seemed to have suffered more from both divorce and widowhood in terms of worsening quality of life and increasing psychological distress. This finding is concordant with previous research on marital transition and mortality^9^. In terms of psychological distress, participants with high educational attainment seemed to have coped with divorce the best but widowhood the worst. This is a new and unexpected finding and may be related to the higher levels of independence, resources and support among those with higher socioeconomic status to cope with an expected traumatic event, such as divorce. Widowhood is less planned and more permanent and may exert severe emotional stress on individuals in the short-term, regardless of skills, resources and support. Divorce had a much stronger impact on depression in men than women, which is consistent with the literature on divorce and mortality^8,9^. It has been documented that men are more likely to dramatically lose supportive social ties^9^ and experience declined social support from their children following a divorce^47^. Finally, interestingly, women who were widowed seemed to have benefited from reduced heavy alcohol consumption. A previous study in France found that women decreased heavy drinking prior to and at the time of widowhood^48^. Some evidence suggests that husbands may influence wives’ drinking behaviour^49^, it is plausible that the death of a husband may be associated with reduced drinking occasions.

### Limitations

The current study is the first to our knowledge to examine short- and longer-term effects of marital disruption on a broad range of physical, psychological and behavioural health outcomes in middle-aged and older adults. Strengths include a population-based sample, comprehensive proximal health outcomes, and examination of both divorce and widowhood. However, findings should be interpreted in light of limitations. First, some relevant information was not collected by the 45 and Up Study, such as relationship quality, the exact time of marital transition (we could only infer that the event happened between T1 and T2), the long-term cumulative marital history (e.g., the total number of marriages and broken relationships)^35^. Such information is important to further elucidate whether the adverse health effects of marital disruption are due to social selection or causation. While this study focused on marital disruption, the other type of marital transition, namely remarriage could further affect health behaviours and outcomes. However, we did not model this transition because of the small number of participants who remarried and the lack of repeated measures to ascertain long-term effects of remarriage. Second, there was some evidence for selection bias as those who became divorced or widowed by T2 were more likely to become lost to follow-up by T3 (Supplementary file). Third, the number of participants who became divorced or widowed during the study follow-up was small, limiting the power of detecting potential associations and effect modification. Fourth, the 45 and Up Study cohort was not population representative and participants were on average healthier than the general population. However, a study comparing the current cohort with a population representative sample in New South Wales found the estimates for the associations between risk factors and health outcomes to be similar, despite the differences in risk factor prevalence^50^. Finally, it is important to note that the current study was conducted based on a sample aged 45 years and above and we only examined the effects of marital disruption in midlife and at an older age. Findings may not generalise to younger populations.

## Conclusions

This current Australian study extends previous evidence on marital transition and health and suggests that marital disruption can be a vulnerable life stage, particularly for certain subgroups, such as men. Findings from the study have important public health implications. Given the ubiquitous and inevitable nature of marital disruption, it is important to raise public awareness of its potential health effects and develop strategies to help individuals navigate such difficult life transitions. Physicians and other health practitioners who have access to regularly updated patient information may play an important role in identifying at-risk individuals, monitoring their health and referring them to potential interventions and support programs.

## Supplementary Information

Supplementary Information.

## Data Availability

The data that support the findings of this study are available from the Sax Institute upon application and payment, which were used under license for the current study, and so are not publicly available. Data are however available from the authors upon reasonable request and with permission of the Sax Institute.
