# Supplementary material for: Effects of divorce and widowhood on subsequent health behaviours and outcomes in a sample of middle-aged and older Australian adults
Source: Sci Rep. 2021 Aug 2;11:15237. doi: 10.1038/s41598-021-93210-y (PMC8328969; doi:10.1038/s41598-021-93210-y)
Supplement: Supplementary file 1 — Supplementary Information. [file 41598_2021_93210_MOESM1_ESM.pdf]

Effects of divorce and widowhood on subsequent health behaviours and outcomes in a sample of middle-aged and older Australian adults

Ding Ding,[1,2*] melody.ding@sydney.edu.au

Joanne Gale, [1,2] joanne.gale@sydney.edu.au

Adrian Bauman, [1,2] adrian.bauman@sydney.edu.au

Philayrath Phongsavan, [1,2] philayrath.phongsavan@sydney.edu.au

Binh Nguyen[1,2] thanh-binh.nguyen-duy@sydney.edu.au

[1]Prevention Research Collaboration, Sydney School of Public Health, Faculty of Medicine and Health, The University of Sydney, Camperdown, New South Wales, Australia

[2]Charles Perkins Centre, The University of Sydney, Camperdown, New South Wales, Australia

***Corresponding author:**

Ding Ding, 6N69 Charles Perkins Centre (D17), the University of Sydney, Camperdown, New South Wales, 2006, Australia

melody.ding@sydney.edu.au

Supplementary files

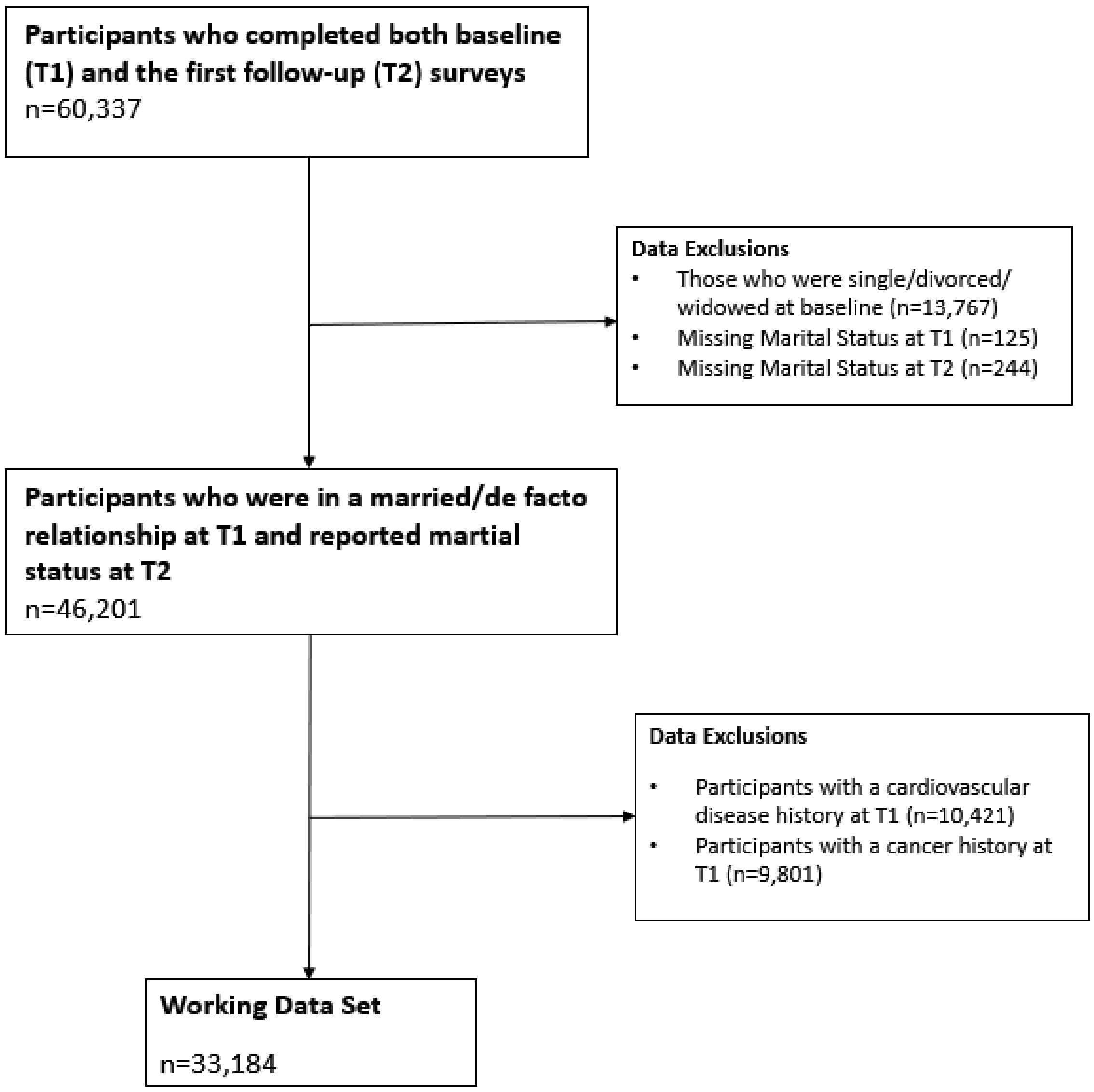

Supplementary Figure 1: Flow diagram participants in Analysis 1

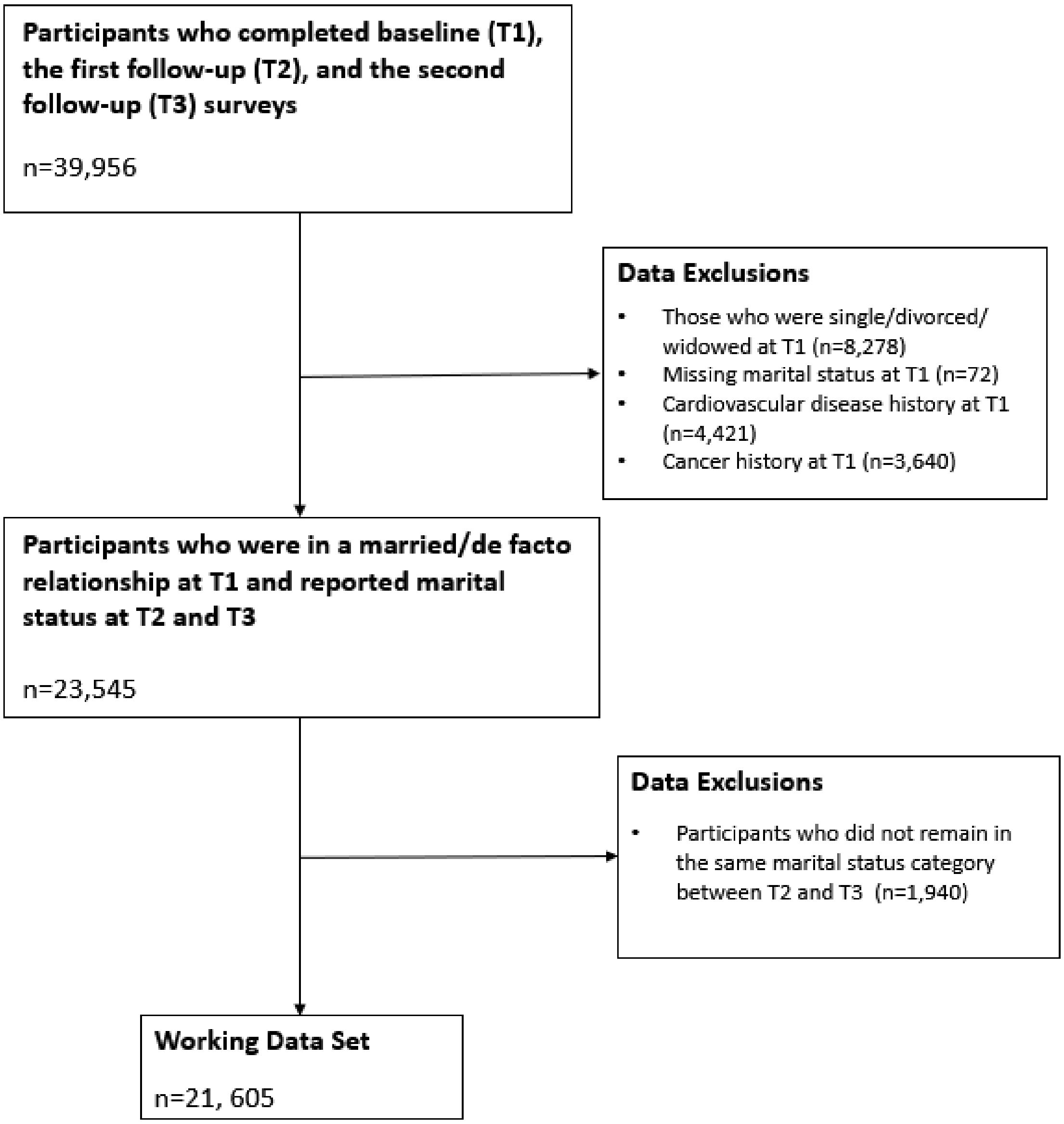

Supplementary Figure 2: Flow diagram participants in Analysis 2

Supplementary Table 1. Baseline (T1, 2006-2009) characteristics of participants by marital transition status between T1, T2 (2010), and T3 (2012-2016)

Characteristics	Overall (n=21,605)	Continuously married (n=20,900)	Remained divorced (n=270)	Remained widowed (n=435)
	n (%)	n (%)	n (%)	n (%)
**Sociodemographic characteristics**				
Age: Mean (SD)	58.4 (8.32)	58.2 (8.19)	56.0 (8.10)	67.2 (9.65)
Sex (female)	11,561 (53.5)	11,078 (53.0)	150 (55.6)	333 (76.6)
Education				
Low (up to 10 years)	5,335 (24.9)	5,091 (24.6)	60 (22.3)	184 (42.8)
Mid (high school/diploma/trade)	9,182 (42.9)	8,888 (42.9)	119 (44.2)	175 (40.7)
High (degree)	6,889 (32.2)	6,728 (32.5)	90 (33.5)	71 (16.5)
Living in major cities	10,548 (49.8)	10,236 (49.9)	124 (47.1)	188 (43.8)
Born overseas	4,682 (21.7)	4,526 (21.7)	61 (22.6)	95 (21.8)
**Baseline health behaviours and outcomes**				
Fair/poor self-rated health	1,050 (5.0)	1,006 (4.9)	22 (8.4)	22 (5.3)
Fair/poor quality of life	675 (3.2)	630 (3.1)	18 (7.1)	27 (6.5)
High Kessler 10 score (K10≥22)	829 (4.1)	792 (4)	23 (9)	14 (3.7)
Self-reported anxiety diagnosis	1,079 (7.6)	1,047 (7.5)	16 (9.7)	16 (7.4)
Self-reported depression diagnosis	1,645 (11.5)	1,589 (11.4)	36 (21.8)	20 (9.2)
Smoking	934 (4.3)	892 (4.3)	25 (9.3)	17 (3.9)
Alcohol ≥ 14 serves/week	4,354 (20.4)	4,209 (20.4)	67 (25.3)	78 (18.2)
Physical inactivity	3,420 (16.1)	3,289 (16.0)	48 (18.3)	83 (19.7)
Insufficient fruit and vegetable intake	16,258 (76.9)	15,743 (76.9)	209 (79.2)	306 (72.7)

T1: baseline data collection (2006-09); T2: first follow-up: 2010; T3: second follow-up: 2012-16.

Methodological supplement

Overall, 71% of participants in Analysis 1 provided complete follow-up data at T3. People who became divorced or widowed by T2 may have deferential risk of death or loss-to-follow-up by T3. This may pose threats for selection bias. If participants' health deterioration following marital disruption has led to their loss-to-follow-up (particularly as a result of death) at T3, then our findings from Analysis 2 are likely to have biased towards the null as a result of "survival bias". To address this, we flagged participants who were lost to follow-up for any reason between T2 and T3, and particularly those who had lost to follow-up because of death (based on linkage data from the NSW Registry of Births, Deaths, and Marriages). We ran logistic regression models to test the relationship between marriage transition categories between T1 and T2 and death or loss-to-follow-up by T2 and T3, adjusted for all covariates specified in the main models.

Our findings suggest that when adjusted for all covariates, those who became divorced or widowed between T1 and T2 were more likely to have lost to follow-up at T3 (OR [95%CI]=1.57 [1.33-1.86] for divorce and OR [95%CI]=1.23[1.07-1.43] for widowhood) but they were not more likely to have died before T3 (OR [95%CI]=1.34 [0.54-3.31] for divorce and OR [95%CI]=1.11[0.67-1.83] for widowhood). This additional analysis suggests that selection bias is likely to have affected Analysis 2. However, given that there was no evidence for higher odds of death among those who became divorced or widowed, the reason for loss-to-follow-up may not be health related (e.g., address change). Therefore, it is unclear in which direction loss-to-follow-up could have biased our findings from Analysis 2.

STROBE Statement—Checklist of items that should be included in reports of ***cohort studies***

	Item No	Recommendation
**Title and abstract**	1	(*a*) Indicate the study's design with a commonly used term in the title or the abstract<br>Most the title and the abstract indicate that this is a prospective study.
		(*b*) Provide in the abstract an informative and balanced summary of what was done and what was found<br>We described a balance summary in the abstract.
**Introduction**		
Background/rationale	2	Explain the scientific background and rationale for the investigation being reported<br>We provided scientific background and rationale clearly in the Introduction (Pages 3-4)
Objectives	3	State specific objectives, including any prespecified hypotheses<br>We outlined the research objectives in the last paragraph of the Introduction (Pages 4-5)
**Methods**		
Study design	4	Present key elements of study design early in the paper<br>We described key elements of study design clearly in the Methods section. (Page 5)
Setting	5	Describe the setting, locations, and relevant dates, including periods of recruitment, exposure, follow-up, and data collection<br>We provided all this information in the Methods section under "Study population" (Page 5)
Participants	6	(*a*) Give the eligibility criteria, and the sources and methods of selection of participants. Describe methods of follow-up<br>We provided all this information in the Methods section under "Study population" (Page 5)
		(*b*) For matched studies, give matching criteria and number of exposed and unexposed<br>Not applicable
Variables	7	Clearly define all outcomes, exposures, predictors, potential confounders, and effect modifiers. Give diagnostic criteria, if applicable<br>We provided all this information in the Methods section under "Measures" and in Table 1 (Pages 6-7)
Data sources/ measurement	8*	For each variable of interest, give sources of data and details of methods of assessment (measurement). Describe comparability of assessment methods if there is more than one group<br>We provided all this information in the Methods section under "Measures" and in Table 1 (Pages 6-7)
Bias	9	Describe any efforts to address potential sources of bias<br>Selection bias tested and described in Supplementary file (Page 5 Supplementary file)
Study size	10	Explain how the study size was arrived at<br>We described exclusion under "exposure variable" and present participant flow charts om Supplementary files (Page 6-7)
Quantitative variables	11	Explain how quantitative variables were handled in the analyses. If applicable, describe which groupings were chosen and why<br>Described in Table 1.
Statistical methods	12	(*a*) Describe all statistical methods, including those used to control for confounding<br>Described under "Statistical analysis" (Page 8)

		(*b*) Describe any methods used to examine subgroups and interactions<br>Multiplicative interactions tested and methods described under "Statistical analysis" (Page 8)
		(*c*) Explain how missing data were addressed<br>Missing data were minimal and missing as category approach was used. Described under "Statistical analysis" (Page 8)
		(*d*) If applicable, explain how loss to follow-up was addressed<br>We included an additional analysis to address selection bias related to follow-up issue in the supplementary file. Described under "Statistical analysis"
		(*e*) Describe any sensitivity analyses<br>We included an additional analysis to address selection bias related to follow-up issue in the supplementary file (Page 5 Supplementary file). Described under "Statistical analysis" (Page 8)
**Results**		
Participants	13*	(a) Report numbers of individuals at each stage of study—eg numbers potentially eligible, examined for eligibility, confirmed eligible, included in the study, completing follow-up, and analysed<br>Presented in supplementary figures.
		(b) Give reasons for non-participation at each stage<br>Presented in supplementary figures.
		(c) Consider use of a flow diagram<br>Presented in supplementary files, page 1 and 2.
Descriptive data	14*	(a) Give characteristics of study participants (eg demographic, clinical, social) and information on exposures and potential confounders<br>Described under "Baseline descriptive statistics" and Table 2
		(b) Indicate number of participants with missing data for each variable of interest<br>We described the percentage for missing overall, <8%
		(c) Summarise follow-up time (eg, average and total amount)<br>Described under "Baseline descriptive statistics" for Analysis 1 (Page 9-10), and "Analysis 2: Long-term health outcomes following marital dissolution" for Analysis 2 (Page 11)
Outcome data	15*	Report numbers of outcome events or summary measures over time<br>We did not report this as we have too many outcome variables in both analyses , we focused on the associations instead.
Main results	16	(*a*) Give unadjusted estimates and, if applicable, confounder-adjusted estimates and their precision (eg, 95% confidence interval). Make clear which confounders were adjusted for and why they were included<br>Presented in the Results section and Tables 3-5
		(*b*) Report category boundaries when continuous variables were categorized<br>Described in detail in Table 1
		(*c*) If relevant, consider translating estimates of relative risk into absolute risk for a meaningful time period<br>We think that Odds Ratio is the best parameter to report considering the binary nature of out outcome.
Other analyses	17	Report other analyses done—eg analyses of subgroups and interactions, and sensitivity analyses<br>Presented in Supplementary file Page 4.
**Discussion**		
Key results	18	Summarise key results with reference to study objectives

		Described throughout the Discussion (Pages 12-14)
Limitations	19	Discuss limitations of the study, taking into account sources of potential bias or imprecision. Discuss both direction and magnitude of any potential bias<br>Described under “Limitations” in the Discussion (Pages 16-17)
Interpretation	20	Give a cautious overall interpretation of results considering objectives, limitations, multiplicity of analyses, results from similar studies, and other relevant evidence<br>Described under “Conclusions” (Page 17)
Generalisability	21	Discuss the generalisability (external validity) of the study results<br>Described under “limitations” (Pages 16-17)
**Other information**		
Funding	22	Give the source of funding and the role of the funders for the present study and, if applicable, for the original study on which the present article is based<br>Funding information provided with the submission

*Give information separately for exposed and unexposed groups.

perf

the saxinstitute

THE 45 AND UP STUDY

Research to improve health and wellbeing

The 45 and Up Study
GPO Box 5289
Sydney NSW 2001
Helpline: 1300 45 11 45
www.45andUp.org.au

<TITLE> <FIRST NAME> <LAST NAME>
<ADDRESS LINE 1>
<ADDRESS LINE 2>
<ADDRESS LINE 3>
<STATE> <POSTCODE>

<DATE>

Dear <FIRST NAME>,

The 45 and Up Study
The SEEF Project

The 45 and Up Study is a long-term health study of over 250,000 people in NSW aged 45 and over. In <MONTH> <YEAR> you agreed to participate in the 45 and Up Study by completing a questionnaire about your health and lifestyle and signing a consent form. By allowing the 45 and Up Study to follow your health over time you are contributing to a better understanding of the major causes of disease and disability in mid to later life. Thank you very much for joining us.

As a participant in the 45 and Up Study, we now seek your involvement in the SEEF Project – a research project that extends the original study to look at how Social, Economic and Environmental Factors (SEEF) contribute to healthy ageing. This project is very large scale, with 100,000 people being asked to assist us, and SEEF will add important information to the 45 and Up Study to help us gain a better understanding of the factors that influence health and wellbeing.

On the other side of this page is an invitation from Professor Adrian Bauman of the School of Public Health at the University of Sydney to participate in the SEEF Project.

If you would like to participate, please complete the questionnaire, sign the consent form, and return it to us in the provided reply paid envelope. More information about the SEEF Project and how to participate is provided in the information pamphlet. You can tear off this letter to keep for your records if desired.

Taking part is entirely your decision, and, if you choose to take part, you can withdraw at any time by calling the 45 and Up Study Helpline on 1300 45 11 45. Your choice will not affect the health care or benefits you receive, or disadvantage you in any way.

Yours sincerely

Professor Emily Banks
Scientific Director, The 45 and Up Study

In partnership with

Cancer Council New South Wales | Heart Foundation | NSW HEALTH | NSW Government Human Services Ageing, Disability & Home Care | beyondblue | UnitingCare Ageing

perf

TO BE OVER PRINT mailing info needs to be in 10pt to fit barcode and potential 5 line address in order to gain maximum discount all info must be clearly visible in window with specified clearances around barcode

perf

THE UNIVERSITY OF SYDNEY

Invitation to join the SEEF Project

Your name has been randomly drawn from the 45 and Up Study participants' list. As a participant in the 45 and Up Study, we now invite you to join the Social, Economic and Environmental Factors (SEEF) Project.

Participation is as easy as completing the confidential questionnaire that follows this letter, signing the consent form giving your permission for us to use the information you have provided, and returning it to us as soon as possible using the enclosed reply paid envelope.

Your participation in this project will only take about 40 minutes of your time and will make a significant contribution to the SEEF Project, which is examining the factors that help our population stay healthy as it ages.

All information given by you to the 45 and Up Study and the SEEF Project will remain completely confidential and used for health research only.

More information about the SEEF Project and the 45 and Up Study more generally is given in the enclosed information pamphlet and on the Study website (www.45andUp.org.au) or you can call the 45 and Up Study Helpline on **1300 45 11 45**. The information pamphlet and this letter can be kept for your own records.

We very much hope that you will be able to take part.

Thank you.

Yours sincerely

Professor Adrian Bauman
Sesquicentenary Professor of Public Health
School of Public Health

perf

Reflex Blue Pantone 346

THE 45 AND UP STUDY
Research to improve health and wellbeing

THE UNIVERSITY OF SYDNEY

The SEEF Project

Social, Economic and Environmental Factors Questionnaire

The 45 and Up Study relies on the willingness of its participants to share information about their lives and experiences, to provide knowledge that will help people live healthy and fulfilling lives for as long as possible.

This questionnaire looks at which social, economic and environmental factors play a key role in the health and wellbeing of people in the 45 and Up Study.

Participation is completely voluntary, and you are free to withdraw at any time. To take part, please read the participant information leaflet, then complete the questionnaire and consent form and return them in the envelope provided. We very much hope you will be able to take part.

Any questions or comments? Please call the Study Helpline: **1300 45 11 45** or go to **www.45andUp.org.au**

Your answers and experiences are important to us.

To help us read your answers, please use a BLACK or BLUE pen.

Put crosses ☒ OR numbers 7 2 in the appropriate box(es).

General questions about you

1. **What is today's date?**
D D / M M / Y Y Y Y

2. **What is your date of birth?**
D D / M M / Y Y Y Y

3. **What is your gender?** male ☐ female ☐

4. **Have you ever been a regular smoker?** Yes ☐ No ☐ → Go to **Q8**

5. **Are you a regular smoker NOW?** Yes ☐ → Go to **Q7** No ☐

6. ***If No*** **– how old were you when you stopped smoking regularly?**
☐☐ *years old*

7. **About how much do you/ did you smoke on average each day?** *(if you are an ex-smoker, how much did you smoke on average when you smoked? Leave blank if it does not apply)*
☐☐ *cigarettes per day* ☐☐ *pipes and cigars per day*

8. **About how many alcoholic drinks do you have each week?**
one drink = a glass of wine, middy of beer or nip of spirits
(put "0" if you do not drink, or have less than one drink each week)
☐☐ *number of alcoholic drinks each week*

9. **On how many days each week do you usually drink alcohol?** *(put "0" if you do not drink alcohol)*
☐☐ *days each week*

Reflex Blue Pantone 346

10. How many TIMES did you do each of these activities LAST WEEK?
(please put "0" if you did not do this activity)

	times in the last week
**Walking continuously, for at least 10 minutes** *(for recreation or exercise or to get to or from places)*	☐☐
**Vigorous physical activity** *(that made you breathe harder or puff and pant, like jogging, cycling, aerobics, competitive tennis, but not household chores or gardening)*	☐☐
**Moderate physical activity** *(like gentle swimming, social tennis, vigorous gardening or work around the house)*	☐☐

11. If you add up all the time you spent doing each activity LAST WEEK, how much time did you spend ALTOGETHER doing each type of activity?
(please put "0" if you did not do this activity)

	hours		minutes
**Walking continuously, for at least 10 minutes** *(for recreation or exercise or to get to or from places)*	☐☐	:	☐☐
**Vigorous physical activity** *(that made you breathe harder or puff and pant, like jogging, cycling, aerobics, competitive tennis, but not household chores or gardening)*	☐☐	:	☐☐
**Moderate physical activity** *(like gentle swimming, social tennis, vigorous gardening or work around the house)*	☐☐	:	☐☐

12. About how much do you weigh?

☐ *kg* **OR** ☐ *stone* ☐ *lbs*

13. About how many serves of vegetables do you usually eat each day? *a serve is half a cup of cooked vegetables or one cup of salad (please include potatoes and put "0" if less than one a day)*

☐☐ *number of serves of cooked vegetables each day*

☐☐ *number of serves of raw vegetables each day (e.g. salad)*

☐ I don't eat vegetables

14. About how many serves of fruit or glasses of fruit juice do you usually have each day? *a serve is 1 medium piece or 2 small pieces or 1 cup of diced or canned fruit pieces (put "0" if you eat less than one serve per day)*

☐☐ *number of serves of fruit each day*

☐☐ *number of glasses of fruit juice each day*

☐ I don't eat fruit

15. About how many HOURS in each 24 hour DAY do you usually spend doing the following?
(please put "0" if you do not spend any time doing it. The total number of hours does not need to add up to 24)

hours per day		hours per day	
☐☐	*sleeping (including at night & naps)*	☐☐	*sitting*
☐☐	*watching television*	☐☐	*standing*
☐☐	*using a computer*	☐☐	*driving*

16. What is your religious faith or group?
(please __cross__ only one box)

- ☐ Buddhism
- ☐ Christianity
- ☐ Hinduism
- ☐ Islam
- ☐ Judaism
- ☐ other religions/faith
- ☐ no religion

17. Rate your agreement with the following statement:

	strongly agree	agree	disagree	strongly disagree
I try hard to carry my beliefs over into all other dealings in life	☐	☐	☐	☐

18. Which of these most closely describes your sexual orientation? *(please __cross__ only one box)*

- ☐ exclusively heterosexual
- ☐ mainly heterosexual
- ☐ bisexual
- ☐ mainly homosexual (lesbian/gay)
- ☐ exclusively homosexual (lesbian/gay)
- ☐ I don't know
- ☐ I don't want to answer

Questions about you and your household

19. What best describes your current situation?
(please __cross__ only one box)

- ☐ single
- ☐ widowed
- ☐ married
- ☐ divorced
- ☐ de facto/living with partner
- ☐ separated

20. Including yourself, how many people in total live in your household? *(put "1" if you live alone)*

☐☐

21. How many financially dependent children do you have aged:
(put "0" if you have no dependents)

☐☐ *under 15 years old?*

☐☐ *15-24 years old?*

22. What best describes your current housing or family home? *(please* ***cross*** *only one box)*

- ☐ house
- ☐ hostel for the aged
- ☐ nursing home
- ☐ flat, unit, apartment
- ☐ mobile home
- ☐ retirement village, self care unit
- ☐ house on farm
- ☐ other

23. Do you (or any other members of this household) own this home, rent it, or do you live here rent free?

- ☐ own
- ☐ currently paying off mortgage/ involved in a rent-buy scheme
- ☐ rent (or pay board)
- ☐ live here rent free/ life tenure

24. Do you currently own (or are paying off mortgage for) any other property that you do not live in? ☐ Yes ☐ No

25. In the past year, at times my house/apartment has felt: *(please* ***cross*** *as many as apply)*

- ☐ so cold that I have had trouble sleeping
- ☐ so hot that I have had trouble sleeping
- ☐ so cold that I have had trouble going about my normal activities
- ☐ so hot that I have had trouble going about my normal activities
- ☐ comfortable
- ☐ none of the above

26. How is your house/apartment cooled? *(please* ***cross*** *only one box)*

- ☐ no cooling system
- ☐ central (ducted) air conditioning
- ☐ room air conditioning
- ☐ evaporative air conditioning

27. What is your MAIN source of drinking water at home? *(please* ***cross*** *only one box)*

- ☐ public water supply
- ☐ bottled water
- ☐ rainwater
- ☐ private bore, spring or well
- ☐ other (specify) ____________
- ☐ other private water supply
- ☐ combination of different water sources

28. During the past 5 years, have you changed your main source of drinking water due to:

	Yes	No
drought?	☐	☐
poor water quality?	☐	☐

29. How many motor vehicles in working order are there at your household?

☐☐ *vehicles*

Questions about your health

30. In general, how would you rate your:

	excellent	very good	good	fair	poor
overall health?	☐	☐	☐	☐	☐
quality of life?	☐	☐	☐	☐	☐
eyesight? (with glasses or contact lenses, if you wear them)	☐	☐	☐	☐	☐
hearing?	☐	☐	☐	☐	☐
memory?	☐	☐	☐	☐	☐
teeth and gums?	☐	☐	☐	☐	☐

31. Do you regularly need help with daily tasks because of long-term illness or disability? *(e.g. personal care, getting around, preparing meals)* ☐ Yes ☐ No

32. Do you ever need someone to help with, or be with you for, self care activities? *(e.g. doing everyday activities such as eating, showering, dressing or toileting)* ☐ Yes ☐ No

33. Do you ever need someone to help with, or be with you for, body movement activities? *(e.g. getting out of bed, moving around at home or at places away from home)* ☐ Yes ☐ No

34. Do you ever need someone to help with or be with you for, communication activities? *(e.g. understanding, or being understood by others)* ☐ Yes ☐ No

35. During the past 12 months, how many times have you fallen to the floor or ground? *(put "0" if you haven't fallen in this time)*

☐☐ *times*

36. Have you had a broken/fractured bone in the last 3 years? ☐ Yes ☐ No → *Go to Q37*

***If YES*, which bones were broken?**

- ☐ wrist
- ☐ ankle
- ☐ other (specify) ____________
- ☐ arm
- ☐ rib
- ☐ hip
- ☐ finger/toe

How old were you when it happened? *(give age at most recent fracture if more than one)*

☐☐ *years old*

Reflex Blue Pantone 346

Reflex Blue Pantone 346

37. Has a doctor EVER told you that you have:
(if YES, please ***cross*** *the box and give your age when the condition was first found)*

	Yes	age when condition was first found
skin cancer *(not melanoma)*	☐	age
melanoma	☐	age
breast cancer	☐	age
prostate cancer *(men only)*	☐	age
other cancer	☐	age
type of cancer *(please describe)*		
heart disease	☐	age
type of heart disease *(please describe)*		
high blood pressure *(women only – when pregnant)*	☐	age
high blood pressure *(women only – when not pregnant)*	☐	age
high blood pressure *(men only)*	☐	age
stroke	☐	age
diabetes	☐	age
blood clot *(thrombosis)*	☐	age
enlarged prostate *(men only)*	☐	age
asthma	☐	age
hayfever	☐	age
depression	☐	age
anxiety	☐	age
Parkinson's disease	☐	age
chronic kidney disease	☐	age
**none of these**	☐	

38. In the last month have you been treated for:
(if YES, please ***cross*** *the box and give your age when the treatment started)*

	Yes	age started treatment
cancer	☐	age
heart attack or angina	☐	age
other heart disease	☐	age
high blood pressure	☐	age
high blood cholesterol	☐	age
blood clotting problems	☐	age
asthma	☐	age
osteoarthritis	☐	age
thyroid problems	☐	age
osteoporosis or low bone density	☐	age
depression	☐	age
anxiety	☐	age
**none of these**	☐	

39. Are you NOW suffering from any other important illness? **Yes** ☐ **No** ☐

please describe this illness and its treatment

40. Have you ever been screened for colorectal (bowel) cancer? **Yes** ☐ **No** ☐

Go to ***Q41***

***If YES*, please indicate which test(s) you had:**

☐ faecal occult blood test *(test for blood in the stool/faeces)*

☐ sigmoidoscopy *(a tube is used to examine the lower bowel; this is usually done in a doctor's office without pain relief)*

☐ colonoscopy *(a long tube is used to examine the whole large bowel; you would usually have to have an enema or drink large amounts of special liquid to prepare the bowel for this)*

What year did you have the most recent one of these tests? (*eg.* 2005) Y Y Y Y

41. **About how many times a week are you usually troubled by leaking urine?**

- ☐ never
- ☐ once a week or less
- ☐ 2-3 times
- ☐ 4-6 times
- ☐ every day

42. **Do you regularly care for a sick or disabled family member or friend?** Yes ☐ No ☐ → *Go to Q44*

If YES, **about how much time each week do you usually spend caring for this person?**

☐ full time **OR** [ ][ ] *hours/wk*

43. **Do you live with the person you care for?** Yes ☐ No ☐

44. **Does your health now LIMIT YOU in any of the following activities?**

	Yes, limited a lot	Yes, limited a little	No, not limited at all
VIGOROUS activities (e.g. running, strenuous sports)	☐	☐	☐
MODERATE activities (e.g. pushing a vacuum cleaner, playing golf)	☐	☐	☐
lifting or carrying shopping	☐	☐	☐
climbing several flights of stairs	☐	☐	☐
climbing one flight of stairs	☐	☐	☐
walking one kilometre	☐	☐	☐
walking half a kilometre	☐	☐	☐
walking 100 metres	☐	☐	☐
bending, kneeling or stooping	☐	☐	☐
bathing or dressing yourself	☐	☐	☐

45. **During the past 4 weeks, have you:**

	Yes	No
accomplished less than you would like in your regular daily activities because of your physical health?	☐	☐
been limited in your regular activities because of your physical health?	☐	☐
accomplished less than you would like as a result of any emotional problems?	☐	☐
not done work or other regular activities as carefully as usual as a result of any emotional problems?	☐	☐

46. **During the past 4 weeks, how much did pain interfere with your normal work, including outside the home and housework?**

- ☐ not at all
- ☐ slightly
- ☐ moderately
- ☐ quite a bit
- ☐ extremely

47. **How much time during the past 4 weeks:**

	none of the time	a little of the time	some of the time	most of the time	all of the time
have you felt calm and peaceful?	☐	☐	☐	☐	☐
did you have a lot of energy?	☐	☐	☐	☐	☐
have you felt down?	☐	☐	☐	☐	☐
have your physical health or emotional problems interfered with your social activities?	☐	☐	☐	☐	☐

48. **During the past 4 weeks, about how often did you feel:**

	none of the time	a little of the time	some of the time	most of the time	all of the time
tired out for no good reason?	☐	☐	☐	☐	☐
nervous?	☐	☐	☐	☐	☐
so nervous that nothing could calm you down?	☐	☐	☐	☐	☐
hopeless?	☐	☐	☐	☐	☐
restless or fidgety?	☐	☐	☐	☐	☐
so restless that you could not sit still?	☐	☐	☐	☐	☐
depressed?	☐	☐	☐	☐	☐
that everything was an effort?	☐	☐	☐	☐	☐
so sad that nothing could cheer you up?	☐	☐	☐	☐	☐
worthless?	☐	☐	☐	☐	☐

49. **Thinking of times when you want to see a particular doctor in your practice or medical centre, how quickly do you usually get to see that doctor?**

- ☐ same day
- ☐ next day
- ☐ 2-3 days
- ☐ 4-5 days
- ☐ more than 5 days

50. **Thinking of times when you are willing to see ANY doctor in your practice or medical centre, how quickly do you usually get seen by any doctor?**

- ☐ same day
- ☐ next day
- ☐ 2-3 days
- ☐ 4-5 days
- ☐ more than 5 days

51. **If you need to see a GP urgently, can you normally get seen on the same day?** Yes ☐ No ☐
(leave blank if question does not apply)

Reflex Blue Pantone 346
02-10 SAX SEEF Survey V9 08-02-10

Reflex Blue Pantone 346

52. **In the past 2 years, have you needed to see any specialist doctors?**

☐ Yes ☐ No → *Go to Q54*

53. **After learning you needed to see a specialist doctor(s), how long on average did you have to wait for an appointment?**

- ☐ no waiting period
- ☐ less than a week
- ☐ 1-2 weeks
- ☐ 3-4 weeks
- ☐ 5-8 weeks
- ☐ more than 8 weeks

54. **How long does it take you to travel to your usual or preferred GP?**

- ☐ < 30 minutes
- ☐ 30-59 minutes
- ☐ 1-2 hours
- ☐ 2-3 hours
- ☐ 3-4 hours
- ☐ 4 or more hours
- ☐ doctor visits me

55. **How long does it take you to travel to the nearest hospital?**

- ☐ < 30 minutes
- ☐ 30-59 minutes
- ☐ 1-2 hours
- ☐ 2-3 hours
- ☐ 3-4 hours
- ☐ 4 or more hours

56. **In the past 12 months, about how much have you and your household spent out-of-pocket for medical treatments or services that were NOT covered by Medicare or private insurance?**
(include costs for prescription medicines and tests or treatments by a doctor or health professional, including any gap payment or any items not covered)

- ☐ none
- ☐ $1-$250
- ☐ $251-$500
- ☐ $501-$1,000
- ☐ $1,001-$2,000
- ☐ more than $2,000

57. **In the past 12 months, have you:**

	Yes	No
received a blood pressure check?	☐	☐
had your cholesterol checked?	☐	☐
had your skin checked for cancer?	☐	☐
had a blood test to check your sugar levels?	☐	☐
been told by your GP to eat fewer high fat or high cholesterol foods?	☐	☐
been told by your GP to eat more fruits & vegetables?	☐	☐
been told by your GP to be more physically active?	☐	☐
been told by your GP to quit smoking?	☐	☐

58. **On a scale from 1 to 10, how confident are you that you can do all the things necessary to manage your health on a regular basis?** *(**cross** one number only)*

1	2	3	4	5	6	7	8	9	10
☐	☐	☐	☐	☐	☐	☐	☐	☐	☐

not at all confident … **totally confident**

59. **On a scale from 1 to 10, how confident are you that you can judge when the changes in your health mean you should visit a doctor?** *(**cross** one number only)*

1	2	3	4	5	6	7	8	9	10
☐	☐	☐	☐	☐	☐	☐	☐	☐	☐

not at all confident … **totally confident**

Questions about work and income

Some of these questions ask for details of your economic circumstances. We are asking these because economic and financial factors are important for health.

Remember you do not have to answer any questions if you do not want to and all information will be kept strictly confidential and used for health related research only.

60. **What is your usual yearly HOUSEHOLD income before tax, from all sources?** *(please include wages benefits, pensions, superannuation, etc)*

- ☐ less than $5,000
- ☐ $5,000-$9,999
- ☐ $10,000-$19,999
- ☐ $20,000-$29,999
- ☐ $30,000-$39,999
- ☐ $40,000-$49,999
- ☐ $50,000-$59,999
- ☐ $60,000-$69,999
- ☐ $70,000-$79,999
- ☐ $80,000-$89,999
- ☐ $90,000-$119,999
- ☐ $120,000-$149,999
- ☐ $150,000 or more
- ☐ I would rather not answer this question

61. **What is your usual PERSONAL yearly income before tax, from all sources?** *(please include wages, benefits, pensions, superannuation etc)*

- ☐ less than $5,000
- ☐ $5,000-$9,999
- ☐ $10,000-$19,999
- ☐ $20,000-$29,999
- ☐ $30,000-$39,999
- ☐ $40,000-$49,999
- ☐ $50,000-$59,999
- ☐ $60,000-$69,999
- ☐ $70,000-$79,999
- ☐ $80,000-$89,999
- ☐ $90,000-$119,999
- ☐ $120,000-$149,999
- ☐ $150,000 or more
- ☐ I would rather not answer this question

62. **What is your current work status?**
*(you can **cross** more than one box)*

- ☐ in full time paid work
- ☐ in part time paid work
- ☐ completely retired/ pensioner
- ☐ partially retired
- ☐ disabled/sick
- ☐ self-employed
- ☐ doing unpaid work
- ☐ studying
- ☐ looking after home/family
- ☐ unemployed
- ☐ other

63. **About how many HOURS each WEEK do you and your partner usually spend in paid work?**
(please put "0" if you do not spend any time doing it)

YOU	YOUR PARTNER
hours per week paid work ☐☐	*hours per week paid work* ☐☐
	☐ I do not have a partner

64. **If you are partially or completely retired or not working for another reason, how old were you when you retired or stopped work?** ☐☐ *years old*

Why did you retire/stop work?
*(you can **cross** more than one box)*

- ☐ reached usual retirement age
- ☐ to care for sick/disabled person
- ☐ to look after home/family
- ☐ made redundant
- ☐ could not find a job
- ☐ was self-employed & business closed
- ☐ lifestyle reasons
- ☐ work-related injury
- ☐ own ill health
- ☐ to travel
- ☐ to study
- ☐ other

65. **If you retired due to your own ill health, what was the main health condition leading to your retirement?**
*(please **cross** only one box)*

- ☐ arthritis
- ☐ asthma
- ☐ back problems
- ☐ cancer
- ☐ diabetes
- ☐ digestive system diseases
- ☐ diseases of the nervous system *(e.g. Parkinson's disease)*
- ☐ eye diseases *(e.g. cataracts)*
- ☐ other condition *(specify)*:
- ☐ heart disease/stroke
- ☐ injury – work-related
- ☐ injury – non-work related
- ☐ mental disorders
- ☐ respiratory system diseases
- ☐ other musculoskeletal condition *(e.g. osteoporosis)*

	Yes	No	No partner
66. **Is your partner retired?**	☐	☐	☐

***If Yes,* age s/he retired?** ☐☐ *age*

67. **Over the last 12 months did any of the following happen to you because of a shortage of money?**

	Yes	No
could not fill or collect a prescription medicine	☐	☐
could not get a medical test, treatment, or follow-up that was recommended by a doctor	☐	☐
limited how much fruit and/or vegetables you eat	☐	☐
could not pay electricity, gas or telephone bills on time	☐	☐
could not pay the mortgage or rent on time	☐	☐
asked for financial help from friends or family	☐	☐

Questions about social support

68. **Rate how often the following occur:**

	never	rarely	some times	often	always	N/A*
Your partner makes you feel cared for	☐	☐	☐	☐	☐	☐
Your partner makes too many demands on you	☐	☐	☐	☐	☐	☐
Other family members make you feel cared for	☐	☐	☐	☐	☐	☐
Other family members make too many demands on you	☐	☐	☐	☐	☐	☐
Friends make you feel cared for	☐	☐	☐	☐	☐	☐
Friends make too many demands on you	☐	☐	☐	☐	☐	☐

**** N/A – Not Applicable or do not have partner/family members/friends***

Reflex Blue Pantone 346
02-10 SAX SEEF Survey V9 08-02-10

Reflex Blue Pantone 346

69. Rate how often the following occur:

	never	rarely	some times	often	always	N/A*
Co-workers make you feel cared for	☐	☐	☐	☐	☐	☐
Co-workers make too many demands on you	☐	☐	☐	☐	☐	☐
Co-workers express interest in how you are doing	☐	☐	☐	☐	☐	☐
Co-workers criticise you	☐	☐	☐	☐	☐	☐
Co-workers create tensions or have arguments with you	☐	☐	☐	☐	☐	☐

** N/A – Not Applicable or not in working environment*

70. How often do you feel rushed or pressed for time?

never	rarely	some times	often	always
☐	☐	☐	☐	☐

71. How many TIMES in the LAST WEEK did you:
(please put "0" if you did not spend any time doing it)

	times in the last week
spend time with friends or family who do not live with you?	☐☐
talk to someone (friends, relatives or other personal calls) on the telephone?	☐☐
go to meetings of social clubs, religious groups or other groups you belong to?	☐☐
spend time in internet social activities? *(e.g. social network sites)*	☐☐

72. How many people outside your home, but within one hour of travel, do you feel you can depend on or feel very close to?

☐☐ *people*

73. In the past 12 MONTHS have any of the following been a problem for you or anyone close to you?
(you can ***cross*** *more than one box)*

- ☐ serious illness
- ☐ serious accident
- ☐ serious disability
- ☐ mental illness
- ☐ divorce or separation
- ☐ not able to get a job
- ☐ involuntary loss of job
- ☐ alcohol or drug-related problems
- ☐ gambling problem
- ☐ witness to violence
- ☐ abuse or violent crime
- ☐ trouble with the police
- ☐ death of a family member or close friend
- ☐ none of these

Questions about your neighbourhood

74. Answer YES or NO to the following questions:

	Yes	No
do you go outside your local area to visit your family?	☐	☐
can you get help from friends when you need it?	☐	☐
if you were caring for someone and needed to go out for a while, would you ask a neighbour for help?	☐	☐
have you visited a neighbour in the past week?	☐	☐
when you shop in your local area are you likely to run into friends and acquaintances?	☐	☐
in the past 6 months, have you done a favour for a sick neighbour?	☐	☐
do you agree that most people in your neighbourhood can be trusted?	☐	☐
does your area have a reputation for being a safe place?	☐	☐

75. Rate your agreement with the following statement:

	strongly agree	agree	disagree	strongly disagree
many shops, stores, markets or other places to buy things I need are within easy walking distance of my home	☐	☐	☐	☐
a public transport stop (such as a bus or train) is within a 10-15 minute walk from my home	☐	☐	☐	☐
there are footpaths on most of the streets in my neighbourhood	☐	☐	☐	☐
my neighbourhood has several free or low cost recreation facilities, such as parks, walking paths, swimming pools, etc	☐	☐	☐	☐

75. *continued.* **Rate your agreement with the following statements:**

	strongly agree	agree	disagree	strongly disagree
the crime rate in my neighbourhood makes it unsafe to go on walks at NIGHT	☐	☐	☐	☐
the crime rate in my neighbourhood makes it unsafe to go on walks during the DAY	☐	☐	☐	☐

76. How long have you lived at your current address? ☐☐ *years*

77. List the state, postcode or suburb, and time period for your last two places of residence in AUSTRALIA

77a. What was your most recent PREVIOUS residential location?

Postcode: ☐☐☐☐ OR

Suburb/Town: ____________

State/Territory:

☐ ACT ☐ SA
☐ NSW ☐ TAS
☐ NT ☐ VIC
☐ QLD ☐ WA
☐ OVERSEAS

If overseas, country: ____________

How long did you live there?

☐☐ *years* OR ☐☐ *months*

77b. Where did you live IMMEDIATELY before that?

Postcode: ☐☐☐☐ OR

Suburb/Town: ____________

State/Territory:

☐ ACT ☐ SA
☐ NSW ☐ TAS
☐ NT ☐ VIC
☐ QLD ☐ WA
☐ OVERSEAS

If overseas, country: ____________

How long did you live there?

☐☐ *years* OR ☐☐ *months*

78. List the state, the postcode or suburb, and time period of employment for your last two places of employment in AUSTRALIA

78a. What was your most recent PREVIOUS employment location?

Postcode: ☐☐☐☐ OR

Suburb/Town: ____________

State/Territory:

☐ ACT ☐ SA
☐ NSW ☐ TAS
☐ NT ☐ VIC
☐ QLD ☐ WA
☐ OVERSEAS

If overseas, country: ____________

How long did you work there?

☐☐ *years* OR ☐☐ *months*

78b. Where did you work IMMEDIATELY before that?

Postcode: ☐☐☐☐ OR

Suburb/Town: ____________

State/Territory:

☐ ACT ☐ SA
☐ NSW ☐ TAS
☐ NT ☐ VIC
☐ QLD ☐ WA
☐ OVERSEAS

If overseas, country: ____________

How long did you work there?

☐☐ *years* OR ☐☐ *months*

Don't forget to sign the consent form overleaf.

Please return your completed questionnaire in the envelope provided. No stamp is required.

Reflex Blue Pantone 346

02-10 SAX SEEF Survey V4 08-01-09

Reflex Blue Pantone 346

THE 45 AND UP STUDY

Research to improve health and wellbeing

THE UNIVERSITY OF SYDNEY

Consent Form - The SEEF Project

The 45 and Up Study is designed to provide much needed information about how to stay healthy and independent throughout life. As one of over 250,000 45 and Up Study participants, you have already completed a questionnaire about your health and lifestyle.

We are asking for your help again to give additional information about your health and the social, economic and environmental factors that impact on healthy ageing by completing a questionnaire for the SEEF Project.

By signing this consent form you are agreeing to participate in the SEEF Project, as outlined in the participant information leaflet entitled *"The SEEF Project – Information for Participants"*.

Participation in the SEEF Project is entirely voluntary and you may ask questions or withdraw your consent at any time by calling the 45 and Up Study Helpline on 1300 45 11 45.

In signing this consent form, I agree:

- To the 45 and Up Study providing my questionnaire to the researchers of the SEEF Project.
- To the long-term storage and use of the information from my questionnaire for health-related research.
- To the 45 and Up Study combining the new information from my questionnaire with the information I have already given to the 45 and Up Study, as outlined in the participant information leaflet entitled *"The SEEF Project – Information for Participants"* of which I have a copy.

I give my consent on the understanding that:

- My information will only be used for the purposed outlined in the participant information leaflet entitled *"The SEEF Project – Information for Participants"*.
- My information will be kept strictly confidential and will be used for health research only. Research, reports and publications from the 45 and Up Study and the SEEF Project will be based on de-identified information and will not identify any individual taking part.
- My consent will continue to be valid following death or loss of decision-making capacity unless withdrawn by my next of kin or other person responsible. I may withdraw from the 45 and Up Study or the SEEF Project at any time by calling the 45 and Up Study Helpline on 1300 45 11 45.
- My decision as to whether or not to participate in the SEEF Project or any other additional research will not disadvantage me or affect my future healthcare in any way.
- I may be contacted again by the 45 and Up Study regarding future research, and my participation in this will be entirely voluntary.

I have read all of the above, as well as the information provided in the participant information leaflet entitled *"The SEEF Project – Information for Participants"*. I have been given the opportunity to ask questions and have been fully informed about the SEEF Project. I agree to participate in the SEEF Project.

Name (Print): ______

Signature: ______ *Date today:* D D / M M / Y Y Y Y

Extra contact details:

It would be very helpful and reduce study costs if we could contact you in future by email. If you are happy for us to do this, please print your email address here:

Email address: ______

Have you changed your address? Please let us know your new details:

Surname: ______

Given name(s): ______

Postal address: ______

Town or Suburb: ______ *State or Territory:* ______ *Postcode:* ______

Thank you very much for filling in the questionnaire.

If you have any questions, please ring the 45 and Up Study Helpline on **1300 45 11 45.**

You can also write directly to:
Professor Emily Banks, Scientific Director
The 45 and Up Study
GPO Box 5289, Sydney NSW 2001

Please send your questionnaire (no stamp required) to:
Confidential
The 45 and Up Study
The SEEF Project
Reply Paid 1005
BROADWAY NSW 2007
